# A clinical prediction rule for diagnosing human infections with avian influenza A(H7N9) in a hospital emergency department setting

**DOI:** 10.1186/s12916-014-0127-0

**Published:** 2014-08-05

**Authors:** Qiaohong Liao, Dennis K M Ip, Tim K Tsang, Bin Cao, Hui Jiang, Fengfeng Liu, Jiandong Zheng, Zhibin Peng, Peng Wu, Yang Huai, Eric H Y Lau, Luzhao Feng, Gabriel M Leung, Hongjie Yu, Benjamin J Cowling

**Affiliations:** Division of Infectious Disease, Key Laboratory of Surveillance and Early-warning on Infectious Disease, Chinese Center for Disease Control and Prevention, 155# Changbai Road, Beijing, 102206 P.R. China; Division of Community Medicine and Public Health Practice, School of Public Health, Li Ka Shing Faculty of Medicine, The University of Hong Kong, Hong Kong Special Administrative Region, China; Division of Epidemiology and Biostatistics, School of Public Health, Li Ka Shing Faculty of Medicine, The University of Hong Kong, Hong Kong Special Administrative Region, China; Beijing Chao-Yang Hospital, Beijing Institute of Respiratory Medicine, Capital Medical University, Beijing, China; China-US Collaborative Program on Emerging and Re-emerging Infection Disease, US Centers for Disease Control and Prevention, Beijing, China

**Keywords:** Avian influenza A(H7N9), Clinical prediction rule, Clinical diagnosis, Hospital emergency setting

## Abstract

**Background:**

Human infections with avian influenza A(H7N9) virus are associated with severe illness and high mortality. To better inform triage decisions of hospitalization and management, we developed a clinical prediction rule for diagnosing patients with A(H7N9) and determined its predictive performance.

**Methods:**

Clinical details on presentation of adult patients hospitalized with either A(H7N9)(n = 121) in China from March to May 2013 or other causes of acute respiratory infections (n = 2,603) in Jingzhou City, China from January 2010 through September 2012 were analyzed. A clinical prediction rule was developed using a two-step coefficient-based multivariable logistic regression scoring method and evaluated with internal validation by bootstrapping.

**Results:**

In step 1, predictors for A(H7N9) included male sex, poultry exposure history, and fever, haemoptysis, or shortness of breath on history and physical examination. In step 2, haziness or pneumonic consolidation on chest radiographs and leukopenia were also associated with a higher probability of A(H7N9). The observed risk of A(H7N9) was 0.3% for those assigned to the low-risk group and 2.5%, 4.3%, and 44.0% for tertiles 1 through 3, respectively, in the high-risk group. This prediction rule achieved good model performance, with an optimism-corrected sensitivity of 0.93, a specificity of 0.80, and an area under the receiver-operating characteristic curve of 0.96.

**Conclusions:**

A simple decision rule based on data readily obtainable in the setting of patients’ first clinical presentations from the first wave of the A/H7N9 epidemic in China has been developed. This prediction rule has achieved good model performance in predicting their risk of A(H7N9) infection and should be useful in guiding important clinical and public health decisions in a timely and objective manner. Data to be gathered with its use in the current evolving second wave of the A/H7N9 epidemic in China will help to inform its performance in the field and contribute to its further refinement.

**Electronic supplementary material:**

The online version of this article (doi:10.1186/s12916-014-0127-0) contains supplementary material, which is available to authorized users.

## Background

Human infections with novel avian-origin influenza A(H7N9) virus were first identified in March 2013, [1,2] mainly in the eastern provinces of China [3]. A total of 131 human infections, dominated by severe illness and mortality, were confirmed in the spring wave in mainland China from March through May 2013 [4,5]. With the adoption of suitable public health measures including closure of live poultry markets, few cases were identified over the summer months [6]. However, H7N9 has resurged in this winter, 2013-2014 [7].

In the context of preparing and responding to further waves of this evolving epidemic, the importance of early detection, diagnosis, isolation and reporting of A(H7N9) infections has been repeatedly emphasized [8]. Accurate and objective risk prediction can help physicians to guide clinical management and inform triage decisions for optimizing the utilization of valuable clinical and public health resources that may easily be overwhelmed during an epidemic. However, no simple and reliable decision tool has yet been available for predicting the risk of A(H7N9) influenza in an objective and timely manner.

Our objective in this study was to develop a clinical prediction rule that would accurately identify patients with A(H7N9) influenza on their first presentation to a hospital emergency setting and to evaluate the predictive performance of this rule.

## Methods

We analyzed two databases that contained clinical and basic laboratory data from two groups of patients presenting similarly with acute respiratory infections to hospitals in China, including 121 laboratory-confirmed A(H7N9) cases and 2,603 patients who suffered from acute respiratory infections other than A(H7N9) influenza. Outpatient clinics or emergency departments in hospitals represent a typical first step for patients with acute respiratory infections in China to present to the healthcare system, owing to the coverage of national health insurance programs and the lack of standalone primary healthcare clinics in either the public or private sector as an alternative [9].

The A(H7N9) database consisted of patients who presented clinically with symptoms of acute respiratory infection to hospitals in different provinces of China and were subsequently confirmed to have A(H7N9) virus infection between March and May 2013. In China, all laboratory-confirmed infections with A(H7N9) viruses are reported to the Chinese Center for Disease Control and Prevention (China CDC, Beijing, China) through a national surveillance system [10]. Diagnostic confirmation of A(H7N9)virus infection was done either by the isolation of A(H7N9) virus or a positive real-time reverse-transcription polymerase chain reaction (RT-PCR) assay for A(H7N9) in a respiratory specimen [10]. Case definitions, surveillance for identification of influenza A(H7N9) cases, and laboratory test assays are described in previous reports [10].

The second database consisted of patients who presented similarly with acute respiratory infections to hospitals in Jingzhou city, Hubei province of China from 4 January 2010 through 30 September 2012. Their clinical details were captured by a surveillance program for severe acute respiratory infection (SARI) conducted by China CDC in four surveillance hospitals in Jingzhou during that period [11]. All patients admitted to these four surveillance hospitals were screened by either a nurse or physician. A patient was defined as having SARI if they had on their clinical presentation an elevated temperature (rectal or axillary temperature ≥37.3°C) and at least one sign or symptom of acute respiratory illness, including cough, sore throat, tachypnea, difficulty breathing, abnormal breath sounds on auscultation, sputum production, hemoptysis, chest pain or chest radiograph consistent with pneumonia. Nasopharyngeal swabs were collected from these patients and tested for seasonal influenza and avian influenza H5N1 viruses by RT-PCR. As these samples were all collected before the first H7N9 human case was identified, they were not tested for A(H7N9) virus.

For patients included in either of these databases, clinical information on the medical history and physical examination findings on their initial clinical presentation was abstracted retrospectively from their original medical records using a standard data abstraction sheet by trained nursing and medical officers at each hospital. Patients younger than 14 years of age from both databases were not included in this study, as there are few patients <14 years of age with confirmed A(H7N9) virus infection, and their presenting symptoms, the approach of symptom ascertainment, overall clinical course and disease severity, disease epidemiology, and even health care seeking pattern and pathway were generally very different from that in adults and elderly persons [3–5].

Additional information collected included age, sex, history of any poultry exposure, including exposure to live poultry markets within two weeks of symptom onset, influenza vaccination history, smoking history, pregnancy, history of underlying medical conditions, height (m), weight (kg) and clinical symptoms.

The results of investigations performed on presentation included chest radiography (signs of consolidation and pneumonia), simple hematologic blood analysis including hemoglobin level (Hgb) and leukocyte count (WBC), and C-reactive protein (CRP). Because we did not have sufficiently complete data (>60%) on some laboratory determinations for the entire sample (including platelet count, other serum biochemistry tests and clotting profile), we excluded these determinations from the analysis for all patients.

### Ethics statement

The collection of data from confirmed A(H7N9) cases was determined by the Chinese National Health and Family Planning Commission as part of public health investigations of emerging influenza outbreaks and was exempted from institutional review board assessment. Collection of data of SARI cases was approved by the ethical review committee of the China CDC. Therefore, informed consents from the cases were not required.

### Statistical analysis

A two-step regression model was used to develop the prediction rule, so as to simulate the decision making process in the setting of a clinic or hospital emergency room where a patient first presents [12,13]. In step 1, basic variables obtainable from the medical history and physical examination were employed to identify a subgroup of patients who were more likely to have A(H7N9) and, therefore, needed additional investigation and work-up. Variables used in this stage included age group (<60 years, ≥60 years), sex, poultry exposure history, influenza vaccination history, smoking history, and history of underlying chronic diseases, pregnancy, major presenting symptoms and physical findings. In step 2, simple radiologic and laboratory variables were added to significant predictors from step 1 (those having a *P*-value less than 0.05) to further refine the identification of subgroups having higher risk for A(H7N9). We used a univariate logistic regression model to identify significant (*P* ≤ 0.05) predictors of a final diagnosis of A(H7N9) and then entered them into a multivariable logistic regression model with backward selection. We removed variables that had a *P*-value greater than 0.05. Interaction terms were tested as candidate variables, but none of these terms entered the final models. We derived each prediction rule using all available information on patients. We used multiple imputations [14,15] (20 imputations) in the derivation process to make the most of all available non-missing data while preserving the uncertainty from the missing data in the results [16,17] [see Additional file 1: Appendix].

A score-based prediction rule for a final diagnosis of A(H7N9) was then developed for each step from the final logistic regression equations using a regression coefficient-based scoring method [13]. A simple integer-based point score was assigned for each predictor variable, which was calculated by dividing the corresponding β-coefficients by the absolute value of the smallest coefficient in the final model and rounding up to the nearest integer. The overall risk score for each patient was calculated by adding the scores for each component together [18]. Aiming to be used as a screening tool to capture as many of the A/H7N9 cases as possible, a cutoff was specified with *a priori* sensitivity of 0.99 in step 1 and 0.95 for the overall model (steps 1 and 2).

Total risk scores above the cutoff threshold were then categorized in tertiles as a risk prediction rule for ease of clinical implementation. Performance of the risk prediction rule in predicting A(H7N9) infection was examined by sensitivity, specificity, likelihood ratios for both positive and negative test results, and area under the receiver-operating characteristic (ROC) curve [19]. Calibration was evaluated by using the Hosmer–Lemeshow chi-square statistic (*P* > 0.05 for all models) [20]. Regression models were tested for possible overfitting by using linear shrinkage estimators [13,21].

The prediction rule was internally validated with samples of the same size resampled with replacement from the original derivation data set using the bootstrap method [22]. The model was refitted as the original model derivation process on these bootstrap samples for 1,000 iterations [13,21] to determine the degree of performance deterioration to be expected when applied on an independent sample of patients [21]. We also estimated the optimism-corrected estimates to correct for the absolute magnitude of bias for each performance index [23]. We performed all analyses with R software, version 3.0.1.

## Results

As of the end of May 2013, 131 confirmed A(H7N9) cases were officially reported in mainland China. Among these patients, 121 patients of 14 years of age or older presenting with acute respiratory infection to an emergency department and requiring hospitalization for medical reasons were included in this study [5]. Two cases younger than 14 and eight cases having mild disease and confirmed by routine testing through sentinel influenza-like illness surveillance were excluded from the present study [9].

During the surveillance period from 4 January 2010 through 30 September 2012, 90,890 patients had been hospitalized in the four surveillance hospitals in Jingzhou city. Among these, 25,406 (28%) patients met the SARI case definition within 24 hours of hospital admission. Ninety percent (22,777) were <14 years of age and excluded from the present study. Among the 2,603 included patients, 2,310 (89%) had a nasopharyngeal swab specimen collected, and 430 (19%) tested positive for influenza viruses by rRT-PCR, including 258 (60%) with influenza A(H3N2), 36 (8%) with A(H1N1) pdm09 and 136 (32%) with influenza B.

Table 1 shows the demographic and clinical characteristics of these two groups of patients on presentation to the emergency hospital. On univariate analysis, factors that were associated with an increased risk of A(H7N9) infection included older age (≥60 years), male sex, history of poultry exposure, smoking history, history of underlying medical conditions, fever, cough, haemoptysis, shortness of breath and diarrhea (Table 1). In step 1 of the clinical prediction rule, male sex and history of poultry exposure were independently associated with a final diagnosis of A(H7N9) on multivariable analysis. The presence of three respiratory symptoms, namely fever, haemoptysis and shortness of breath, were also independently associated with a final diagnosis of A(H7N9) (Table 2). Other factors became insignificant (age group, underlying medical illnesses, cough and diarrhea) and were not retained. Fifty-eight percent of the cohort with a total score less than the threshold of 43 was assigned to the low-risk group and did not proceed to step 2 [see Additional file 2: Figure S1].

**Table 1:** **Demographic and clinical characteristics of patients**

**Characteristic**	**H7N9**	**Non-H7N9**	***P*** **-value**
	**(number = 121)**	**(number = 2,603)**	
Demographic			
Age			
14 to 59 years	50 (41.3)	1,293 (49.7)	0.077
≥60 years	71 (58.7)	1,310 (50.3)	
Data missing, %	0 (0)	0 (0)	
Sex			
Female	35 (28.9)	1,045 (40.1)	0.013
Male	86 (71.1)	1,558 (59.9)	
Data missing, %	0 (0)	0 (0)	
History of poultry exposure			
Yes	85 (70.2)	53 (2)	<0.001
No	36 (29.8)	1,583 (60.8)	
Data missing, %	0 (0)	967 (37.1)	
Influenza vaccination history			
Yes	0 (0)	32 (1.2)	0.999
No	36 (29.8)	1,603 (61.6)	
Data missing, %	85 (70.2)	968 (37.2)	
Smoking history			
Yes	26 (21.5)	365 (14)	0.004
No	78 (64.5)	2,238 (86)	
Data missing, %	17 (14)	0 (0)	
History of underlying medical conditions			
Yes	42 (34.7)	846 (32.5)	0.110
No	62 (51.2)	1,751 (67.3)	
Data missing, %	17 (14)	6 (0.2)	
Presence of symptoms on presentation			
Fever	95 (78.5)	1,462 (56.2)	<0.001
Data missing, %	17 (14)	0 (0)	
Cough	95 (78.5)	2,013 (77.3)	<0.001
Data missing, %	17 (14)	3 (0.1)	
Hemoptysis	25 (20.7)	96 (3.7)	<0.001
Data missing, %	17 (14)	11 (0.4)	
Shortness of breath	62 (51.2)	391 (15)	<0.001
Data missing, %	17 (14)	9 (0.3)	
Sore throat	7 (5.8)	418 (16.1)	0.088
Data missing, %	40 (33.1)	10 (0.4)	
Rhinorrhea	2 (1.7)	131 (5)	0.435
Data missing, %	40 (33.1)	9 (0.3)	
Vomiting	4 (3.3)	67 (2.6)	0.351
Data missing, %	17 (14)	9 (0.3)	
Diarrhea	10 (8.3)	34 (1.3)	<0.001
Data missing, %	17 (14)	9 (0.3)	
Investigations			
Chest radiography			
Normal	95 (78.5)	1,908 (73.3)	<0.001
Pneumonia/consolidation	7 (5.8)	690 (26.5)	
Data missing, %	19 (15.7)	5 (0.2)	
Hemoglobin level			
Low	20 (16.5)	903 (34.7)	0.029
Normal	77 (63.6)	1,463 (56.2)	
High	6 (5)	77 (3)	
Data missing, %	18 (14.9)	160 (6.1)	
Leukocyte count			
Low	48 (39.7)	201 (7.7)	0.232
Normal	51 (42.1)	1,567 (60.2)	
High	5 (4.1)	728 (28)	
Data missing, %	17 (14)	107 (4.1)	
C-reactive protein			
Normal	8 (6.6)	192 (7.4)	0.095
High	83 (68.6)	1,025 (39.4)	
Data missing, %	30 (24.8)	1,386 (53.2)

**Table 2:** **Multivariable predicators of a diagnosis of A(H7N9) infection and associated risk scoring system for step 1**

**Characteristic**	***β*** **regression coefficient (95%** **CI)**	**Risk score assigned**
Sex		
Female	Reference	0
Male	0.821 (0.217, 1.425)	10
History of poultry exposure		
Yes	4.259 (3.606, 4.913)	52
No	Reference	0
Presence of symptoms on presentation		
Hemoptysis	1.537 (0.701, 2.372)	19
Shortness of breath	1.961 (1.383, 2.539)	24
Fever	2.624 (1.747, 3.501)	32

Hosmer-Lemeshow statistic, 2.11 (*P* = 0.98). Cutoff threshold for total point score (with a prespecified sensitivity of 0.95): ≥43 indicates high-risk groups; <43 indicates low-risk group. CI, confidence interval.

In step 2, two additional laboratory findings including pneumonia/consolidation on chest radiograph and leukopenia (WBC <4,000/μl) were each independently associated with an increased risk of A(H7N9). On the other hand, finding of leukocytosis (WBC >11,000/μl), or an abnormally low (Hgb < 12 g/dl) or high hemoglobin level (Hgb ≥16 g/dl) were each independently associated with a decreased risk of A(H7N9). All of the previous five factors entered in step 1 had remained statistically significant after adding these two factors. No statistical evidence of overfitting, as demonstrated by linear shrinkage estimation (shrinkage factor, 0.981 (95% confidence interval (CI), 0.976 to 0.984) for step 1 and 0.967 (95% CI, 0.960 to 0.972) for step 2), was seen in either multivariable regression model. For the ease of use in a setting of a clinical consultation, the magnitude of association of each of these factors with A(H7N9)virus infection was quantified by a point scoring system as shown in Table 3. A total score of 68 or greater would indicate the presence of a high risk for A(H7N9) infection, with a prespecified sensitivity of 95% overall. Forty-five percent of patients considered in step 2 were further assigned to the low-risk category [see Additional file 2: Figure S1].

**Table 3:** **Multivariable predicators of a diagnosis of A(H7N9) infection and associated risk scoring system for step 2**

**Characteristic**	***β*** **regression coefficient (95%** **CI)**	**Risk score assigned**
Sex		
Female	Reference	0
Male	0.839 (0.189, 1.489)	11
History of poultry exposure		
Yes	4.028 (3.320, 4.736)	54
No	Reference	0
Presence of symptoms on presentation		
Hemoptysis	1.107 (0.109, 2.105)	15
Shortness of breath	2.240 (1.564, 2.917)	30
Fever	2.769 (1.790, 3.748)	37
Chest radiography		
Normal	Reference	0
Pneumonia/consolidation	1.711 (0.643, 2.778)	23
Leukocyte count		
Low	1.638 (0.909, 2.366)	22
Normal	Reference	0
High	−1.581 (-2.677, -0.485)	−21
Hemoglobin level		
Low	−0.884 (-1.626, -0.143)	−12
Normal	Reference	0
High	−0.075 (-1.739, 1.590)	−1

Hosmer-Lemeshow statistic, 3.57 (*P* = 0.89). Cutoff threshold for total point score (with a prespecified sensitivity of 0.95): ≥68 indicates high-risk groups; <68 indicates low-risk group. CI, confidence interval.

**Figure 1:**
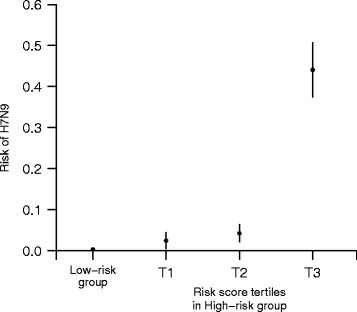
**Risk of influenza A(H7N9) infection stratified by risk categories.** Note: Tertile 1 represents a risk score of 68 to 70, tertile 2 represents a risk score of 71 to 90, tertile 3 represents a risk score of >90.

The magnitude of the scores had good diagnostic utility. When stratified by tertiles with the two cut-points of 70 and 90, a gradation with increasing level of risk for A(H7N9) infection was demonstrated. The corresponding risk of A(H7N9) infection was 0.3% (95% CI, 0.0% to 0.6%) for those assigned to the low-risk group (in steps 1 or 2), 2.5% (95% CI, 0.5% to 4.5%) for tertile 1 (risk score, 68 to 70), 4.3% (95% CI, 2.2% to 6.4%) for tertile 2 (risk score, 71 to 90) and 44.0% (95% CI, 37.4% to 50.8%) for tertile 3 (risk score >90) in the high-risk group. A similar gradation of risk was also observed in the validation analysis (Figure 1).

**Figure 2:**
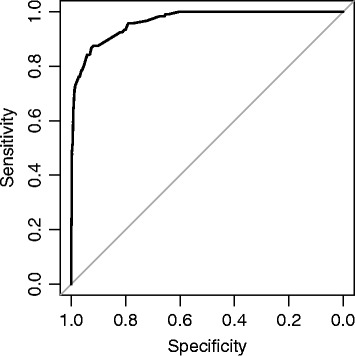
**Receiver-operating characteristic (ROC) curve of the risk prediction rules with different pre-specified level of sensitivity.**

This clinical prediction rule achieved good discriminative ability. It gave a sensitivity of 0.93 and a specificity of 0.80 (optimism-corrected estimates) in the derivation process, which were broadly maintained, respectively, at 0.96 and 0.75 in the bootstrap internal validation process (Table 4). On the other hand, the optimism-corrected area under the ROC curve was 0.96 from both the derivation and the internal validation processes (Figure 2). Likelihood ratios for a positive result with an assignment to the high-risk group after step 2 were moderately strong at 4.62 and 3.85 for the derivation and internal validation processes, respectively. Likelihood ratios for a negative result with assignment to the low-risk group after step 1 or step 2 were 0.090 and 0.056 for the derivation and internal validation processes, respectively.

**Table 4:** **Performance indices for the clinical prediction rule**

**Index**	**Estimate (95%** **CI)**		
	**Derivation indices (n = 2,724)**	**Internal validation by bootstrapping (n = 2,724)**	**Optimism-corrected indices (n = 2,724)**
Sensitivity	0.940 (0.895, 0.984)	0.958 (0.890, 0.994)	0.929 (0.881, 0.954)
Specificity	0.799 (0.783, 0.815)	0.751 (0.614, 0.885)	0.799 (0.783, 0.815)
Likelihood ratio			
Positive test result	4.670 (4.124, 5.309)	3.853 (2.570, 7.747)	4.624 (3.986, 4.887)
Negative test result	0.076 (0.019, 0.134)	0.056 (0.009, 0.125)	0.090 (0.056, 0.147)
Area under ROC curve	0.966 (0.951, 0.981)	0.961 (0.952, 0.966)	0.960 (0.944, 0.976)

Obtained from internal validation by using a bootstrap analysis in which the cohort was resampled 1,000 times with replacement.Likelihood ratio for a positive test result refers to the likelihood of assignment to the high-risk group after step 2. Likelihood ratio for a negative test result refers to the likelihood of assignment to the low-risk group after steps 1 or 2. CI, confidence interval; ROC, receiver-operating characteristic.

## Discussion

Our study presents a decision rule for objectively predicting A(H7N9) infection in adult patients presenting with severe respiratory illness. Factors of particular importance in the prediction rule, including poultry exposure history, fever, shortness of breath and leukopenia, agreed generally with findings reported from previous epidemiological and clinical studies [4]. We had chosen the model with the best performance in terms of both the high sensitivity and area under the ROC curve, which were also maintained in the validation samples, to identify patients having a high risk for the infection at their initial clinical presentation so as to optimize resources during an epidemic.

As generally recognized by previous reports, most laboratory-confirmed cases of A(H7N9) have had a high risk of disease progression and fatality [3,5]. Early initiation of antiviral treatment and provision of a suitable level of intensive care have been identified as important factors in determining the final outcome of patients hospitalized with A(H7N9) virus infection [24]. Our decision tool allows an initial risk assessment to be performed by frontline physicians in a setting where simple laboratory and radiographic examination may not be readily available, based only on simple information obtainable from the history and physical examination. In a setting with greater resources, the risk estimation of those deemed to have a non-trivial risk at step 1 could be further refined by the availability of simple radiographic and laboratory testing results. The scoring system also helps to categorize those being predicted as high-risk into different risk strata to facilitate further clinical decision-making (including the need for further work up, admission decision and ward allocation, initial treatment regimen, level of care and monitoring, and so on) before a definitive diagnosis based on RT-PCR can be available, often at a much later time. Despite the current belief that A(H7N9) virus may not be readily transmitted from person to person, [3,10] the existence of limited person-to-person transmission in a close contact setting [25] also carries an implication for this risk stratification tool to better inform isolation decision and practice.

Depending on resource availability and surge capacity, patients assigned to different risk groups may need to be handled differently. Generally, however, patients assigned to the low-risk group in either steps 1 or 2 should have little risk implication to justify their admission at that particular juncture either for monitoring or hospitalized care unless having other indications for admission. This strategy can help to reduce the demands on inpatient beds and testing capacity. On the other hand, persons having a total risk score exceeding the threshold (≥68) in step 2 should be considered for admission for further assessment and possible initiation of treatment. The provision of different levels of monitoring and treatment to such patients could be guided by the magnitude of the total risk score, which is predictive of the eventual risk of confirmed A(H7N9) infection. Allocation of isolation facilities including individual negative-pressure isolation rooms may also be informed by the individual risk score in case there is any enhancement in human-to-human transmissibility.

Our study included all cases of clinically confirmed A(H7N9) infection that presented with a picture of acute respiratory infection in a hospital setting in the first epidemic in China from March through May. Our control consisted of a suitable sample of patients who clinically presented in a comparable setting within the same geographical area and were captured by a large-scale surveillance network, in a period immediately prior to the A(H7N9) epidemic. Although controls had not been directly tested for A(H7N9) virus, their recruitment in a period during which human A(H7N9) infection was absent or at least very unlikely [26] should have helped to avoid their potential contamination by unascertained A(H7N9) cases.

However, our study does suffer from a number of potential limitations. First, as the H7N9 positive cases and the control patients were not gathered from exactly the same setting, subtle biases may still be introduced by potential variability in the degree of exposure ascertainment or data documentation in the two different settings. Although this problem should have been partly addressed by excluding any variables that were not measured or were generally missing from either group, some measured variables may have been more thoroughly ascertained for A(H7N9) cases, especially in an epidemic setting. Second, a small proportion of all confirmed A(H7N9) cases were either asymptomatic or only mildly symptomatic and had not been hospitalized [9]. Because of the very different clinical picture, our prediction rule may not be applicable for assessing the risk of A(H7N9) infection in this group of patients. Third, as our study had already included almost all confirmed cases of A(H7N9) reported during the initial epidemic in China, the decision rule could only be internally validated by bootstrapping but lacked a suitable sample for external validation. As a result, the actual performance and utility of this prediction rule in future epidemics remains to be determined. Ideally, our rule can be further validated in the current evolving second wave of epidemic in Chinawithin a setting where all cases presenting with acute respiratory infection (ARI) can be captured and ascertained with a definitive testing for A/H7N9 irrespective of their initial risk stratification status. This can inform the assessment of its performance in the field and contribute to its further refinement. Finally, as the prediction rule was derived mainly based on data of the A/H7N9 virus with little or no human-to-human transmission potential, its performance may become very different should the virus acquire the ability to do so. Other factors that may affect performance of the model in future waves of the evolving epidemic may include variation in disease severity, changing risk perception and health care seeking behavior. While the prediction of absolute risk for having A(H7N9) infection could be affected by variation in local factors, robustness of the model for separating higher from lower risk should probably be preserved. When being applied in the field, frontline physicians should remain alert concerning the potential limitations associated with practice guidelines and our decision rule should only supplement, but never supersede, the physician’s judgment in equivocal or borderline cases.

## Conclusions

Our prediction rule currently represents an important evidence-based decision tool for the triage of suspected cases of A(H7N9) infection when they first present clinically in an emergency department or primary care settings. This decision tool will be most useful in an evolving epidemic when the health system’s surge capacity could be overwhelmed by the number of patients seeking care. With the current re-emergence of the A(H7N9) epidemic as the second wave in China, it would be a very timely and practical tool for helping both physicians working on the frontline to make important clinical decisions and public health professionals and health administrators to optimize the proper allocation of limited resources.

## Additional files

Additional file 1
**Appendix.** Details on the multiple imputation and validation processes involved in building the risk prediction model [14–17,23]. Additional file 2: Figure S1. Flowchart of influenza A(H7N9) infection stratified by risk categories.

## Abbreviations

China CDC: Chinese Center for Disease Control and Prevention
CI: confidence interval
CRP: C-reactive protein
Hgb: hemoglobin
ROC: receiver-operating characteristic
RT-PCR: real-time reverse-transcription polymerase chain reaction
SARI: severe acute respiratory infection
WBC: leukocyte count
