# Supplementary material for: A clinical prediction rule for diagnosing human infections with avian influenza A(H7N9) in a hospital emergency department setting
Source: BMC Med. 2014 Aug 5;12:127. doi: 10.1186/s12916-014-0127-0 (PMC4243192; doi:10.1186/s12916-014-0127-0)
Supplement: Additional file 1 — Appendix. Details on the multiple imputation and validation processes involved in building the risk prediction model [14–17,23]. [file 12916_2014_127_MOESM1_ESM.docx]

**Appendix 1**

***Multiple imputation***

To make the most of all available nonmissing data while preserving the uncertainty from the missing data [14-16], we used multiple imputation in the derivation process as follows:

We first simulated 20 imputed data sets before fitting the 2-step regression model. When we performed backward selection, we computed the parameter estimates at every step of selection by computing the parameter estimates from the 20 imputed data and having 20 parameter estimates and using Rubin’s formula to combine them to obtain pooled estimates [17]. The same approach was applied as for univariate analysis to identify significant predictors.

***Validation***

To internally validate our prediction rule and also correct for potential bias due to overfitting in each performance index [23], we conducted the bootstrap validation and correction as follows:

1. Using the original data set, we developed the prediction rule described in the main text, and computed the performance index on the original data set and denoted it as $D_{\mathrm{app}}$.
2. For every bootstrapped data set, we used the same method to develop the prediction rule by using the bootstrap sample and compute the performance index on the bootstrapped data set, denote it as $D_{\mathrm{boot}}$
3. Using the developed prediction rule based on the bootstrapped data, compute its performance index on the original data set, denote it as $D_{\mathrm{orig}}$
4. The overestimation on the performance index is then obtained by $D_{\mathrm{boot}}-D_{orig}$
5. We repeated step 2 to 4 1000 times and hence we get the estimate on the overestimation on the performance index by averaging $D_{\mathrm{boot}}-D_{\mathrm{orig}}$ from the 1000 bootstrapped sample, denote it as OE.
6. The bootstrap corrected performance index is $D_{\mathrm{app}}-OE$

**Appendix References**

14. Schafer JL (1999) Multiple imputation: a primer. Stat Methods Med Res 8: 3-15.

15. Harrell FE J (2001) Regression modeling strategies: with applications to linear models, logistic regression, and survival analysis. New York: Springer Verlag.

16. Little RJA RD (2002) Statistical Analysis With Missing Data: Wiley.

17. Rubin DB (1996) Multiple imputation after 18+ years. Journal of the American Statistical Association 91: 473-489.

23. Harrell FE, Jr., Lee KL, Mark DB (1996) Multivariable prognostic models: issues in developing models, evaluating assumptions and adequacy, and measuring and reducing errors. Stat Med 15: 361-387.
