# Supplementary figures and images for: A clinical prediction rule for diagnosing human infections with avian influenza A(H7N9) in a hospital emergency department setting
Source: BMC Med. 2014 Aug 5;12:127. doi: 10.1186/s12916-014-0127-0 (PMC4243192; doi:10.1186/s12916-014-0127-0)

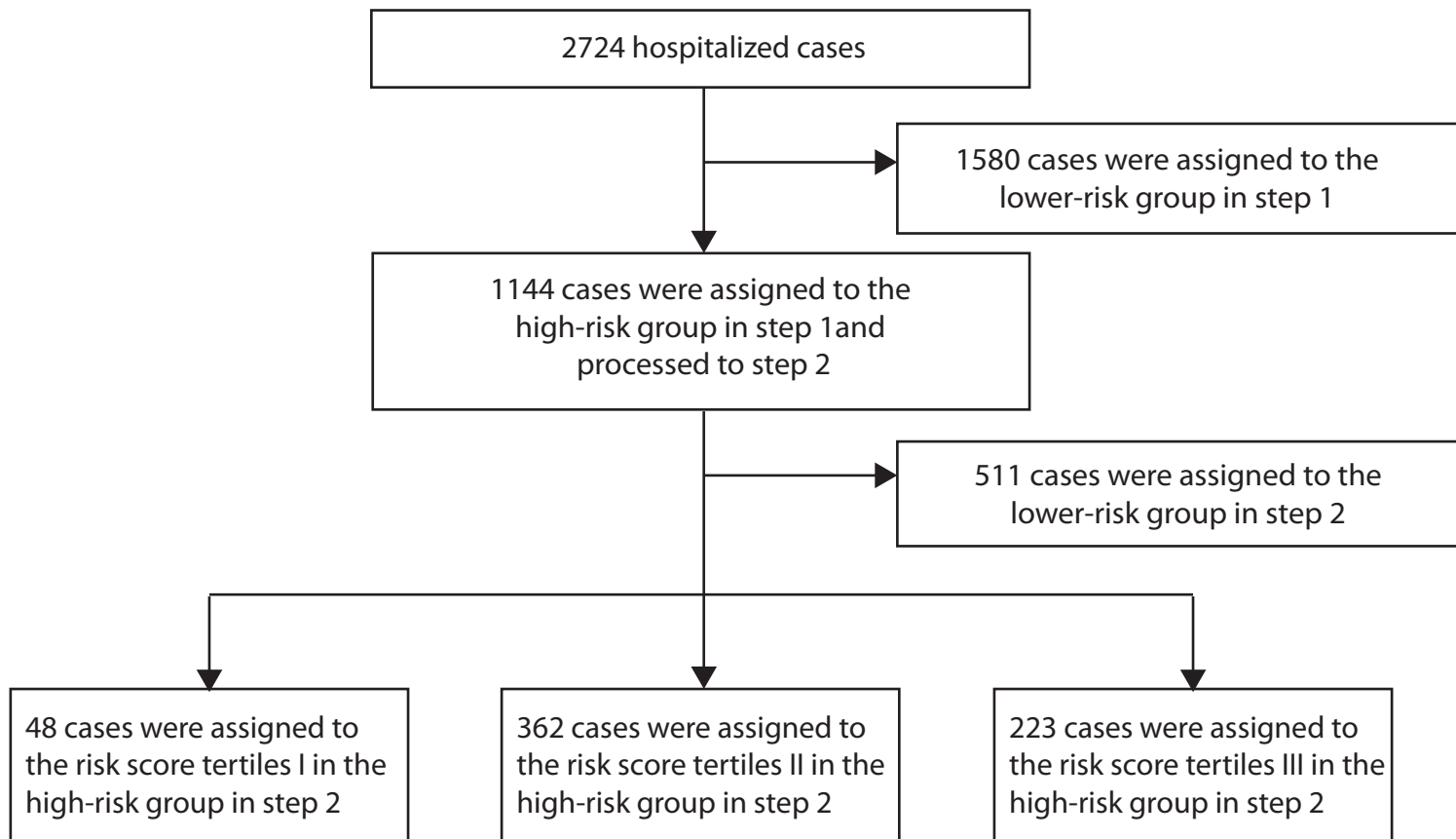

Supplement: Additional file 2: Figure S1. — Flowchart of influenza A(H7N9) infection stratified by risk categories. [file 12916_2014_127_MOESM2_ESM.pdf]
